# Multiple Transcript Properties Related to Translation Affect mRNA Degradation Rates in *Saccharomyces cerevisiae*

**DOI:** 10.1534/g3.116.032276

**Published:** 2016-09-13

**Authors:** Benjamin Neymotin, Victoria Ettorre, David Gresham

**Affiliations:** Department of Biology, Center for Genomics and Systems Biology, New York University, New York 10003

**Keywords:** codon usage, mRNA degradation, 4-thiouracil, translation

## Abstract

Degradation of mRNA contributes to variation in transcript abundance. Studies of individual mRNAs have shown that both *cis* and *trans* factors affect mRNA degradation rates. However, the factors underlying transcriptome-wide variation in mRNA degradation rates are poorly understood. We investigated the contribution of different transcript properties to transcriptome-wide degradation rate variation in the budding yeast, *Saccharomyces cerevisiae*, using multiple regression analysis. We find that multiple transcript properties are significantly associated with variation in mRNA degradation rates, and that a model incorporating these properties explains ∼50% of the genome-wide variance. Predictors of mRNA degradation rates include transcript length, ribosome density, biased codon usage, and GC content of the third position in codons. To experimentally validate these factors, we studied individual transcripts expressed from identical promoters. We find that decreasing ribosome density by mutating the first translational start site of a transcript increases its degradation rate. Using coding sequence variants of green fluorescent protein (GFP) that differ only at synonymous sites, we show that increased GC content of the third position of codons results in decreased rates of mRNA degradation. Thus, in steady-state conditions, a large fraction of genome-wide variation in mRNA degradation rates is determined by inherent properties of transcripts, many of which are related to translation, rather than specific regulatory mechanisms.

Alterations in the abundance of mRNA result from changes in both the rate of transcript synthesis and the rate of transcript degradation. Synthesis and degradation of mRNAs is critical for control of gene expression and cell survival, as ablation of either process results in rapid loss of viability (Nonet *et al.* 1987; Anderson and Parker 1998). The *cis* and *trans* factors that control rates of mRNA synthesis have been studied extensively in many systems (reviewed in Hager *et al.* 2009). By comparison, far less is known about factors that control rates of mRNA degradation. A complete understanding of gene expression regulation requires identification of the sources of variation in mRNA degradation.

Our understanding of the mechanisms by which mRNAs are degraded (reviewed in Parker 2012) is largely the result of studies of specific transcripts (Decker and Parker 1993; Muhlrad *et al.* 1995; Beelman *et al.* 1996). These studies have shown that mRNA degradation is controlled by *cis* factors, including sequence elements in the coding (Parker and Jacobson 1990; Wisdom and Lee 1991) and untranslated (Shaw and Kamen 1986; Muhlrad and Parker 1992) regions, as well as *trans* factors, including RNA binding proteins (Olivas and Parker 2000; Chen *et al.* 2001) and noncoding RNAs (Jing *et al.* 2005). However, the extent to which these different factors impact global patterns of mRNA degradation remains unclear.

Genome-wide mRNA degradation rates have been determined for a number of organisms including bacteria (Selinger *et al.* 2003), plants (Narsai *et al.* 2007), flies (Thomsen *et al.* 2010), mouse (Rabani *et al.* 2011) and human (Duan *et al.* 2013) cell lines. In the budding yeast, *Saccharomyces cerevisiae*, genome-wide mRNA degradation rates have been measured using a variety of methods, including transcriptional inhibition (Wang *et al.* 2002; Grigull *et al.* 2004; Shalem *et al.* 2008), genomic-run-on (García-Martínez *et al.* 2004), and metabolic labeling (Munchel *et al.* 2011; Miller *et al.* 2011). In general, the concordance between different global studies of mRNA degradation rates is poor, likely due to a combination of technical and biological sources of variation. Recently, we introduced RNA Approach to Equilibrium Sequencing (RATE-seq), which combines 4-thiouracil (4-tU) labeling and RNA-seq for determination of genome-wide *in vivo* mRNA degradation rates (Neymotin *et al.* 2014). Using approach to equilibrium labeling kinetics and nonlinear regression, RATE-seq overcomes several problems with existing methods, providing improved accuracy of mRNA degradation rates estimates in steady-state conditions.

Despite discrepancies in estimates of mRNA degradation rates among different studies, three consistent features have been demonstrated across multiple transcriptome-wide datasets. First, there is variation in the rates at which different transcripts are degraded: some vary by as much as an order of magnitude. Second, transcripts for genes encoding functionally related products have similar degradation rates (Selinger *et al.* 2003; Wang *et al.* 2002; Neymotin *et al.* 2014; Yang *et al.* 2003). Third, no single property of transcripts explains the observed variation (Narsai *et al.* 2007; Wang *et al.* 2002; Munchel *et al.* 2011; Miller *et al.* 2011). This latter point suggests that either causative factors are obscured in genome-wide studies, or a combination of different factors affect rates of degradation that have transcript-specific effects. Potential properties of transcripts that might impact their rate of degradation include transcript length (Dressaire *et al.* 2013; Duan *et al.* 2013), GC content (Kudla *et al.* 2009), transcript abundance (Dressaire *et al.* 2013), codon usage (Carlini 2005; Presnyak *et al.* 2015), and folding properties. However, testing the effect of any single property of transcripts on global degradation rates is inherently challenging as each parameter can vary independently across transcripts. At the same time, many transcript properties are correlated with each other, making it difficult to identify causative factors. Thus, to identify determinants of variation in mRNA degradation rates, all known properties of transcripts must be considered simultaneously, and experimental designs that modulate a single property are required for validation experiments.

Here, we analyzed factors that affect mRNA degradation rates in *S. cerevisiae* using multiple regression analysis (Eck and Stephan 2008; Duan *et al.* 2013). We examined the contribution to genome-wide variation in mRNA degradation rates of multiple transcript properties for which genome-wide measurements exist, or can be calculated, including protein levels (Ghaemmaghami *et al.* 2003), protein half-life (Belle *et al.* 2006), RNA abundance (Lipson *et al.* 2009), transcription rates (Pelechano *et al.* 2010), UTR lengths (Nagalakshmi *et al.* 2008), ribosome density (Ingolia *et al.* 2009), association with RNA binding proteins (Hogan *et al.* 2008), the codon adaptation index (CAI) (Sharp and Li 1987), and normalized translational efficiency (nTE) (Pechmann and Frydman 2013). A multiple regression model applied to mRNA degradation rates determined using RATE-seq accounts for 50% of the variation in mRNA degradation rates. Although less variation is explained when multiple regression models are applied to other genome-wide mRNA degradation datasets, many predictors are significant in multiple datasets, suggesting that they are reproducible transcript properties that impact degradation rates. These features include ribosome density, CAI, nTE, and GC content of the wobble position in codons (GC3), suggesting that translation and mRNA degradation rates are interdependent.

Using experimental studies of individual transcripts, we show that decreasing ribosome density of a transcript increases its mRNA degradation rate resulting in decreased steady state transcript levels. Using GFP coding sequence variants that differ only in their GC3 content, we show that coding sequence affects mRNA degradation. Increasing the GC3 content results in decreased mRNA degradation rates and increased steady-state levels. Our experimental results confirm that mRNA degradation rates are determined by multiple factors, many of which are linked to translation.

## Materials and Methods

### Plasmid construction

pCM188 (Garí *et al.* 1997) was used as the backbone for all plasmids. This low copy CEN4 plasmid contains the *URA3* gene, a constitutively expressed tetracycline transactivator, and a multiple cloning site with a *CYC1* TATA region upstream, all under control of two copies of the tetracycline operator. Transcription of the gene is repressed in the presence of tetracycline or its derivatives, including doxycycline. Plasmids DGP148, DGP149, DGP231, and DGP147 are pCM188 with degenerate forms of GFP (Kudla *et al.* 2009), in which the proportion of GC content in the third position of each codon is 0.38 (GFP1), 0.60 (GFP2), 0.67 (GFP3), and 0.71 (GFP4). The coding sequence of each GFP was cloned into the *Bam*HI and *Not*I sites. Plasmid DGP217 is pCM188 with the *GAP1* coding sequence and 3′UTR cloned into the *Bam*HI and *Not*I sites. Plasmid DGP218 contains the same insert as DGP217, except the start codon of *GAP1* was mutated to GTG.

### Strains and growth conditions

The laboratory strain, FY3 (*MATa ura3-52*), which is isogenic to S288C, was used for all experiments. DGY696, DGY697, DGY698, and DGY1281 contain plasmids DGP147, DGP148, DGP149, and DGP231, respectively. DGY1193 and DGY1194 contain plasmids DGP217 and DGP218, respectively, with the *GAP1* locus deleted from start to stop with the KanMX4 cassette.

For all experiments, a single colony for each strain was inoculated in synthetic complete medium without uracil, to maintain selection of the plasmids. Studies of the *GAP1* transcript were performed in nitrogen-limiting medium, with proline as the limiting nitrogen source, as described in Hong and Gresham (2014). Saturated cultures from overnight cultures were back-diluted 1:50 into medium of the same composition. Cells were allowed to grow for 5.5 hr (∼2.5 doublings) before transcription was inhibited with doxycycline at a final concentration of 10 μg/ml. Cells were collected by filtration and snap frozen in liquid nitrogen.

### RNA processing and qRT-PCR analysis

RNA was extracted using the hot acid phenol-chloroform method as in Neymotin *et al.* (2014). Purified RNA was then treated with RQ1 DNAse according to manufacturer recommendations. Reverse transcription was performed using random hexamers and M-MLV reverse transcriptase. Quantitative Reverse Transcription PCR (qRT-PCR) was performed using the SYBR Green system and a Roche Light Cycler. RNA levels were quantified relative to *HTA1*, a housekeeping gene that is unaffected by doxycycline addition. Ratios were calculated using the formula: *Y* = 2^[−(HTA1_ct_ - Gene_^ct^_)]^ where ct is the calculated cycle threshold. RNA levels from each time point were normalized to *t* = 0, which was set to 1. All analyses with error bars are the mean ± SE for 3–6 biological replicates. Values without error bars are the average of two replicates. Primer sequences had amplification efficiencies of at least 95% on RT products. The amplified products for all GFP strains begin between positions 463 and 586 of the transcripts and were between 80 and 120 bp long. The sequences used for qRT-PCR analysis are as follows: *HTA1* (forward: 5′-GCTGGTAATGCTGCTAGGGATA-3′, reverse: 5′-TTACCCAATAGCTTGTTCAATT-3′), *GFP2* and *GFP4* (forward: 5′-TTGCCGGATAACCACTACCT-3′, reverse: 5′-CCTGCTGCAGTCACAAACTC-3′), *GFP3* (forward: 5′-GCCGATAAGCAGAAGAATGG-3′, reverse: 5′-TGTTGATAATGGTCCGCAAG-3′), GFP1 (forward: 5′-CGACCATTACCAGCAGAACA-3′, reverse: 5′-GGGTCCTTTGACAGAGCAGA-3′), *GAP1-ATG*, and *GAP1-GTG* (forward: 5′-TTTGTTCTGTCTTCGTCAC-3′, reverse: 5′-CTCTACGGATTCACTGGCAGCA-3′). Raw data from qRT-PCR experiments are provided in Supplemental Material, Table S3.

### Multiple regression analysis

For multiple regression analysis, we used degradation rate constants (min^−1^), which are approximately normally distributed. To minimize the effects of extreme outliers on regression analysis, we removed mRNA degradation rates > 1.5 times the interquartile range, which resulted in the exclusion of < 5% of all genes for most datasets. Degradation rates for most datasets were calculated as ln(2)/*t*_half-life_, except in Munchel *et al.* (2011) and Neymotin *et al.* (2014), where the effects of dilution as a result of cellular growth was also considered. Transcript counts were from Lipson *et al.* (2009), and estimated based on an assumption of ∼60,000 mRNA/cell (Zenklusen *et al.* 2008). Protein per mRNA was calculated as the values from Ghaemmaghami *et al.* (2003) divided by the values for counts. CAI (Sharp and Li 1987) and nTE (Pechmann and Frydman 2013) was calculated for each transcript based on codon frequency tables in the seqinr package in R (R Core Team 2013) or in Pechmann and Frydman (2013). As many predictors appear log-normal, we log-transformed predictor variables [log_10_(Variable) or log_10_(Variable + 1)], except for GC content of each codon position and nTE, which are approximately normally distributed, and **Δ***G*, which is negative in value. For categorical variables, transcripts were classified as bound by an RNA-binding protein based on data from Hogan *et al.* (2008), and functionally annotated based on GO SLIM files downloaded from the Saccharomyces Genome Database (SGD; http://www.yeastgenome.org/).

In a linear multiple regression model, a parameter of interest is modeled as being dependent on two or more predictors. We consider the degradation rate constant as the modeled variable, and all genome-wide measurements as predictors. To build the model, we followed two separate approaches. In the first approach, we first determined the *P*-value of the pairwise correlation of each predictor with degradation rate. This indicates whether the regression coefficient is significantly different from zero, and whether or not the predictor has any affect on degradation rate. Next we included all predictors that have a *P*-value < 0.05 into the multiple regression models. We then performed stepwise removal of terms by removing sequentially the predictors with the highest *P*-value. The final model is then the reduced model, where only significant terms remained. We obtained the same result using the step function in R, which reduces models based on Akaike’s Information Criterion (AIC). In a second approach, we first calculated the significance of each predictor when it is the only one in the model, as above. We then started adding to the model based on the predictors with the lowest *P*-value. With each additional term, we checked that all of the terms in the model remained significant. If a new term was added and it was not significant, we removed it from the model. If a new term was added and a different existing predictor was no longer significant, we tested models containing either the new predictor or the one that was no longer significant, and retained the one that explained more variation. We did not add terms that were insignificant in the pairwise correlation with degradation rate. Both approaches gave similar results. Model diagnostics suggest that there is no obvious curvature or patterns in terms of increase or decrease in variance as a function of fitted values (Figure S6). There is also minimal curvature in the normal Q-Q plot, suggesting the model follows linearity (Figure S6).

### Data availability

We performed all analyses using R (R Core Team 2013). In addition to custom written functions in R, we also used functions from the following packages: *TeachingDemos*, *Biostrings*, *LSD*, *stringi*, *GeneRfold*, and *seqinr*. The complete dataset is available as an R workspace, along with the code for performing the analysis and generating all figures at the Open Science Framework: https://osf.io/kf4u5/ (doi:10.17605/OSF.IO/KF4U5).

## Results

### Multiple transcript properties affect global mRNA degradation rates

**Figure 1:**
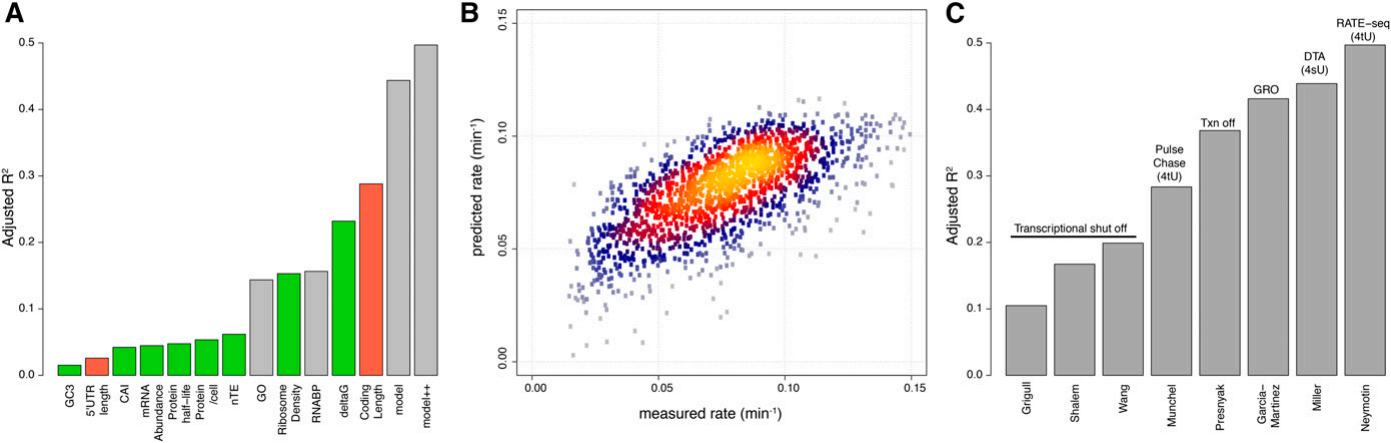
Multiple factors are associated with variation in mRNA degradation rates. (A) Individual predictors explain different amounts of the variation in mRNA degradation rates determined using RATE-seq. Multiple regression models including continuous variables (model), and continuous and categorical variables (model++), explain around half the variation in mRNA degradation rates. Positive correlations with mRNA degradation rates are indicated in red, whereas negative correlations are indicated in green. Values corresponding to ANOVA and multiple regression are in gray. Predictors explaining < 1% of the variation are not shown, but are retained in the final model. (B) A comparison of modeled mRNA degradation rates with measured mRNA degradation rates shows that the model behaves similarly across the entire range of mRNA degradation rates. (C) Multiple regression models applied to other published mRNA degradation rates explain different amounts of the variation. Less variance can be explained for mRNA degradation rates that rely on transcriptional inhibition, with the exception of Presnyak *et al.* (2015). Labeling studies using thiouracil or thiouridine are indicated in parentheses with 4tU and 4sU, respectively. GRO, genomic run on; DTA, dynamic transcriptome analysis.

Previous studies have found evidence for the effect of specific properties of transcripts on the degradation rate of individual transcripts (Shaw and Kamen 1986; Parker and Jacobson 1990; Muhlrad and Parker 1992; Olivas and Parker 2000). We tested the relationship between globally measured transcript features (Table 1) and genome-wide mRNA degradation rates determined using RATE-seq (Table S1). We found that several transcript features are significantly correlated with mRNA degradation rates (Figure 1A and Table S2). The most significant single feature predictive of mRNA degradation rates measured using RATE-seq was the length of the coding sequence, which explains almost 30% of the variance. The folding energy (**Δ***G*) is also significantly associated with mRNA degradation rates, which may be due to the fact that folding energy and coding sequence length are highly correlated. Several features related to the translation of transcripts were also significantly associated with mRNA degradation rates, including ribosome density, the CAI, nTE, and the GC3 content. We also tested whether the function of the encoded product is predictive of mRNA degradation rate, and found that functional assignment using gene ontology (GO) terms explains a significant fraction of the variation (Figure 1A). This is consistent with the observation that transcripts encoding proteins in similar functional categories degrade with similar kinetics (Wang *et al.* 2002; Neymotin *et al.* 2014; Yang *et al.* 2003). In addition, association with specific mRNA-binding proteins also explains a significant fraction of variation in mRNA degradation rates (Figure 1A). These results suggest a relationship between several features of transcripts and the rate at which they are degraded. We observed similar relationships between some predictors and mRNA degradation rates that have been measured using other methods (Figure S1), suggesting that some of these relationships are reproducible despite the poor agreement in mRNA degradation rates among studies.

**Table 1:** Genome-wide parameters included in model testing

Predictor	*N*	Units	Transformation	Reference
Coding length	5850	Nucleotides	log_10_(nucleotides)	SGD*^a^*
3′UTR length	4950	Nucleotides	log_10_(nucleotides+1)	SGD/Nagalakshmi *et al.* 2008
5′UTR length	4345	Nucleotides	log_10_(nucleotides+1)	SGD/Nagalakshmi *et al.* 2008
5′UTR GC content	4345	Proportion	None	SGD/Nagalakshmi *et al.* 2008
3′UTR GC content	4911	Proportion	None	SGD/Nagalakshmi *et al.* 2008
Abundance	5488	Transcripts per million (TPM)	log_10_((TPM/total reads)60,000)	Lipson *et al.* 2009
Protein/cell	3818	Protein/cell	log_10_(protein/cell)	Ghaemmaghami *et al.* 2003
Ribosome density	5269	Reads/kilobase of transcript/million mapped reads (RPKM)	log_10_(RPKM+1)	Ingolia *et al.* 2009
Transcription rate	4346	Molecules/min	log_10_(molecules/min)	Pelechano *et al.* 2010
Coding GC position 1	5850	Proportion	None	SGD
Coding GC position 2	5850	Proportion	None	SGD
Coding GC position 3	5850	Proportion	None	SGD
Protein half-life	3164	Min	log_10_(min)	Belle *et al.* 2006
Δ*G* (minimum free energy of entire transcript)	5850	kcal/mol	None	SGD/GeneRfold
CAI	5850	Relative scale	log_10_(relative scale)	CAI function in R
nTE	5850	Relative scale	None	Pechmann and Frydman 2013
Protein/mRNA	3583	Protein/cell/transcript	log_10_(protein/cell/transcript+1)	Ghaemmaghami *et al.* 2003; Lipson *et al.* 2009

a *S. cerevisiae* Genome Database (http://www.yeastgenome.org/).

Although many transcript properties are correlated with each other (Figure S2), some properties show no correlation and therefore may exert independent and differential effects on the rate of mRNA degradation. Therefore, we used multiple regression analysis to model the contribution of multiple transcript features to variation in mRNA degradation rates simultaneously (*Materials and Methods*). We initially built a model incorporating all factors, and used sequential reduction to arrive at a minimal model (*Materials and Methods*, Figure S7). We find that the explained variation when multiple transcript properties are included exceeds the variance explained by any single factor, suggesting that degradation rates are determined by a combination of transcript features (“model” in Figure 1A). The final model includes the following covariates: coding length, Δ*G*, ribosome density, 5′ UTR length, GC3, nTE, and protein half-life. In addition, it includes GC2 and transcription rate, despite the relatively small contribution of each of these predictors (*i.e.*, individually, they explain < 1% of the variance). When the categorical factors of gene function and association with specific RNA-binding proteins are included, 50% of the variance in mRNA degradation rates can be explained (“model++” in Figure 1A). Thus, rates predicted by a multiple regression model are in good agreement with experimentally determined rates (Figure 1B). Models incorporating all features explain significant fractions of the variation reported in other mRNA degradation datasets, albeit with reduced explanatory power (Figure 1C). Interestingly, we find that, in general, models applied to mRNA degradation rates measured using transcriptional inhibition tend to explain much less variation than models applied to RNA degradation rates measured with metabolic labeling methods.

**Figure 2:**
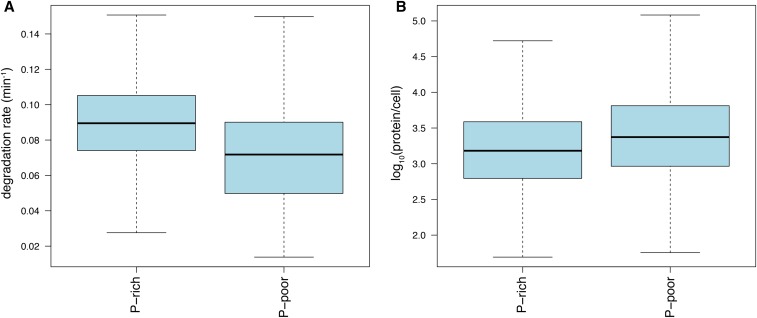
The presence of multiple proline codons affects degradation rates and protein production. (A) Box plots of the mRNA degradation rates of proline-rich and proline-poor proteins. Transcripts that contain sequential proline codons, which slow translation elongation rates, tend to be less stable than other transcripts (Wilcoxon rank sum test, *P* = 7.5 × 10^−32^). (B) The abundance of poly-proline containing proteins is reduced compared to the global distribution of protein abundances.

Our model suggests a negative relationship between mRNA degradation rates and translation elongation rates, as estimated by CAI, nTE, and ribosome density. Translation elongation rates are slowed during peptide bond formation for proline residues (Gardin *et al.* 2014), and particularly when multiple prolines are encoded sequentially. To further investigate the role of translation elongation rates on mRNA degradation, we classified transcripts based on presence or absence of at least four sequential proline codons. Consistent with our multiple regression model, transcripts rich in proline degrade more rapidly than the rest of the transcriptome (Figure 2A). Interestingly, stretches of proline codons are also associated with overall lower protein expression levels (Figure 2B).

### Ribosome density affects mRNA degradation rate

Our computational analysis suggests that different aspects of translation affect mRNA degradation rates. Ribosomes are generally thought to protect mRNAs from degradation (Parker 2012). Consistent with previous analyses (Edri and Tuller 2014), we find that increased ribosome density is associated with decreased rates of mRNA degradation (Figure S3). To experimentally reduce the density of ribosomes on specific transcripts, we mutated the start codon of an endogenous transcript, *GAP1*, which encodes the general amino acid permease, from ATG to GTG, and placed it on a low-copy plasmid under control of doxycycline-repressible promoters (Garí *et al.* 1997). Addition of doxycycline has little affect on cellular physiology, and no detectable affect on global gene expression (Wishart *et al.* 2005). ATG start codons are required for the small ribosomal subunit to recruit the large ribosomal subunit for fully formed ribosomes. Mutation of the *GAP1* start codon to GTG is expected to reduce the number of ribosomes bound to *GAP1* mRNAs as translation initiation at GTG occurs with a frequency of ∼5% compared to ATG start codons (Kolitz *et al.* 2009).

**Figure 3:**
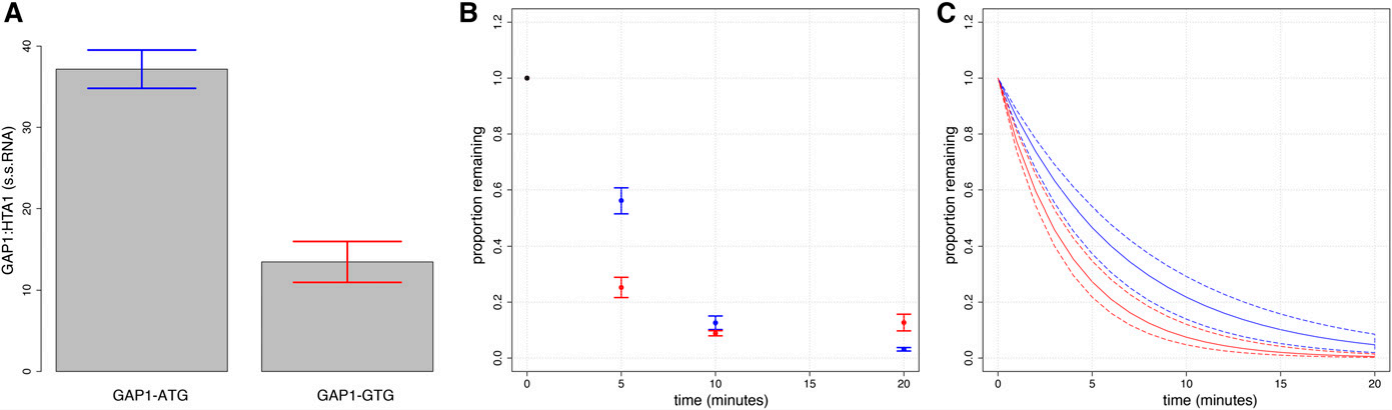
The effect of ribosome density on *GAP1* mRNA degradation rate. (A) Mutation of the wild-type start codon in GAP1 from ATG to GTG results in (A) a reduced steady state transcript level, and (B) an increased mRNA degradation rate. In (B) we show the average value for each time point ± SEM. In (C) we show bootstrapped CI for the regression of all data points. Solid lines indicate the line of best fit, and dotted lines indicate CI. In blue is the transcript with a wildtype start codon. In red is the transcript with a mutated start codon.

We tested the *GAP1* transcripts for alteration in degradation kinetics as a function of start codon mutation. In the absence of the wildtype start codon, we find that the *GAP1* transcript is decreased significantly in steady-state mRNA abundance (Figure 3A), and that the transcript degrades more rapidly upon addition of doxycycline to repress transcription initiation (Figure 3, B and C). This suggests that a decrease in ribosome density results in an increase in the degradation rate of the *GAP1* transcript, consistent with the global trend identified in our computational analysis.

### Decreased GC content of the third codon position increases the rate of mRNA degradation

Our multiple regression model predicts that factors involved in translation, including ribosome density, CAI, nTE, and the GC3 content, contribute to variation in mRNA degradation rates (Figure 1A). GC3 content has been reported to affect mRNA abundance and mRNA degradation in *Escherichia. coli* (Kudla *et al.* 2009). In mammalian cells, GC3 content was also found to affect mRNA levels, but not degradation rates (Kudla *et al.* 2006), implying that mRNA synthesis or processing must underlie differences in mRNA levels. However, a more recent genome-wide study found evidence that decreased GC3 content is correlated with increased mRNA degradation rates (Duan *et al.* 2013).

**Figure 4:**
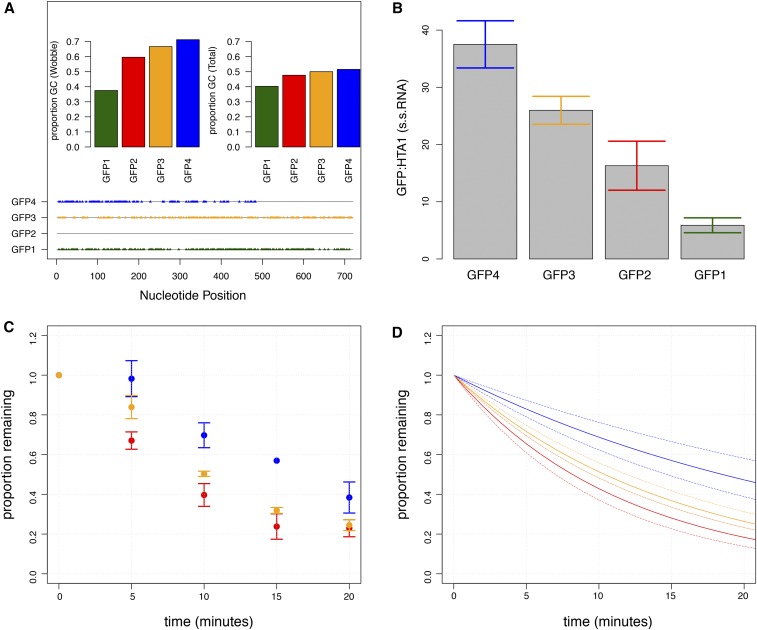
GC3 content affects degradation kinetics and steady state levels. (A) Four GFP transcripts containing synonymous mutations span a range of GC3 content (left) and overall GC (right). Nucleotide-level alignment of each GFP-encoding transcript relative to GFP2. Positions of similarity in sequence are depicted by a gray line, and sites of synonymous mutations are indicated with colored triangles. (B) Differences in GC3 content affect steady state levels of transcripts. (C, D) GFP2 (red), GFP3 (yellow), and GFP4 (blue) degrade in a GC3-dependent manner. In (C) we show the average value for each time point ± SEM. In (D) we show bootstrapped 95% confidence intervals for the regression of all data points. Solid lines indicate the line of best fit, and dotted lines indicate confidence intervals.

To study the contribution of GC3 content to variation in mRNA degradation rates, we used GFP constructs that differ in sequence at synonymous sites only (Kudla *et al.* 2009). We studied four GFP transcripts that span a range of GC3 content from 38 to 71% (Figure 4A). Changes in GC3 content also result in overall variation in total GC content (Figure 4A). Coding sequences were placed under control of the identical doxycycline-regulated promoter on low copy plasmids, and engineered to have the same UTRs. We confirmed that all four constructs result in functional GFP expression (data not shown).

As all coding sequences are expressed from an identical promoter, differences in steady-state mRNA abundance must result from differences in rates of degradation, synthesis, or processing. We find that steady-state mRNA levels vary with GC3 content, with the highest GC3 content resulting in the highest steady-state mRNA abundance (Figure 4B), consistent with observations in mammalian cells (Kudla *et al.* 2006). Following addition of doxycycline to repress transcription initiation, we confirmed that three of the four GFP-encoding transcripts degrade differentially in a GC3-dependent manner (Figure 4, C and D), consistent with our multiple regression prediction. Using the measured steady-state abundances and degradation rates for the three transcripts, we estimated synthesis rates. All three strains have similar estimated rates of synthesis, consistent with differences in degradation rates being the primary determinant of differences in steady-state mRNA levels. We find that a fourth construct, which has much lower GC3 content, and the lowest steady-state abundance, does not significantly differ in its mRNA degradation rate from the second lowest GC3 (Figure S4). This may reflect a limitation of the sensitivity of our assay, or the fact that other factors are likely to interact with the effect of GC3 content.

## Discussion

The abundance of mRNAs is determined by both the rate at which they are synthesized and the rate at which they are degraded. In this study, we sought to construct a comprehensive model that predicts genome-wide variation in mRNA degradation rates. Using multiple regression analysis, we find that 43% of the variation in mRNA degradation rates determined using RATE-seq can be explained by considering multiple properties of transcripts in a single model. By including association with specific RNA-binding proteins and the function of the encoded product, ∼50% of the genome-wide variation in mRNA degradation rates can be explained. Interestingly, we find that, in general, methods for measuring RNA degradation that use transcriptional inhibition tend to explain less variation than less disruptive methods. This may reflect that fact that metabolic labeling methods, which perturb the cell only minimally, yield more physiologically relevant degradation rates than transcriptional inhibition, which results in cell death.

In our analysis of mRNA degradation rates measured using RATE-seq, coding sequence length is the strongest single predictor of mRNA degradation rates: in general, the longer a transcript, the more rapidly it is degraded. Other genome-wide investigations have shown a positive relationship between the length of the mature mRNA and its rate of degradation (Feng and Niu 2007; Dressaire *et al.* 2013; Duan *et al.* 2013; Geisberg *et al.* 2014). In our original study using RATE-seq, we showed that estimates of mRNA degradation rates are not biased by transcript length (Neymotin *et al.* 2014). In the related DTA method of Miller *et al.*(2011), coding length is regressed out of estimates of mRNA degradation, and, therefore, the effect of coding length is no longer apparent in the resulting data. Studies of individual transcripts have shown that increasing transcript length by addition of specific sequences containing “instability elements” enhances a transcript’s rate of degradation (Caponigro *et al.* 1993). Therefore, it is possible that it is not the length of the transcript that affects its degradation, but the presence of additional regulatory elements, to which *trans* factors such as RNA-binding proteins can bind, promoting their degradation.

### Association with ribosomes increases mRNA stability

Our multiple regression analysis provided evidence that protein translation impacts mRNA degradation rates. Previous studies regarding the role of translation initiation rates on mRNA degradation have shown differing results. In a detailed study of the *CYC1* transcript, all start codons were removed from its coding region, thereby preventing ribosome binding and translation (Yun and Sherman 1996). Following global transcriptional inhibition with thiolutin, the untranslated transcript degraded with similar kinetics to the translated transcript. Similarly, when translation of the *MFA2* transcript was inhibited by introducing a strong secondary structure in the 5′ region of the transcript, it was not found to alter the degradation kinetics following transcriptional inhibition using the GAL system (Beelman and Parker 1994). By contrast, using the same method to reduce translation of the *PGK1* transcript results in an increased mRNA degradation rate (Muhlrad *et al.* 1995). Consistent with a recent report (Edri and Tuller 2014), our analysis shows that genome-wide increased association with translation machinery, as measured by ribosome density, is correlated with decreased mRNA degradation rates (Figure 1 and Figure S3).

To validate experimentally the effect of translation initiation rates and ribosome density on the rate of mRNA degradation, we mutated the start codon of *GAP1* from ATG to GTG. Consistent with results from our genome-wide study, loss of the wild-type start codon results in reduced mRNA levels, and an increased rate of mRNA degradation. Using a functional assay for growth inhibition in the presence of d-amino-acids, which are toxic to yeast cells and transported by GAP1, we found that mutation of the start codon to GTG does not result in a complete loss of protein function (Figure S5). This may be attributable to sufficient full-length protein expression from translation initiated at the GTG start codon to confer d-amino-acid sensitivity. In addition, a potential inframe start codon is located 288 nucleotides (96 amino acids) downstream of the wild-type start codon, and its use may result in a protein product that retains much of the wild-type GAP1 activity although the first 96 amino acids of GAP1 are known to affect the functionality and localization of the encoded permease (Merhi *et al.* 2011). An out-of-frame ORF between codons 110 and 187 could potentially promote nonsense mediated decay (NMD) of mutant transcripts. Therefore, in addition to a decrease in ribosome density, we cannot exclude the possibility that alterations in protein function and/or NMD contribute to the decreased stability of the mutant transcript.

We searched for common transcript features to understand why a subset of the transcripts (∼4%) fit the model poorly (>2 SD greater than the mean residual value) (Figure S8). We find that the outlier transcripts, which are degraded more rapidly than expected, are shorter, have lower ribosome density, and are less translationally efficient (based on normalized translational efficiency score). Thus, although shorter transcripts tend to be more stable, the properties of these outliers suggest that reduced translation of short transcripts results in faster degradation, consistent with our overall model.

### Synonymous coding mutations affect mRNA degradation rates

Regression analysis suggested genome-wide relationships between codon usage and mRNA degradation rates. We find a negative correlation between two different measures of biased codon usage, CAI and nTE, and mRNA degradation rate. Bias toward more frequent codons in a transcript may increase the elongation rate during translation (Plotkin and Kudla 2011). Therefore, this negative correlation suggests that faster elongation by ribosomes may result in decreased rates of mRNA degradation. This observation is consistent with the recent report by Presnyak *et al.* (2015), who showed that increased occurrence of optimized codons, as defined by nTE, in transcripts, results in decreased degradation rates. We note that, although the effect of nTE on mRNA degradation rates is significant, it explains only ∼7% of the genome-wide variance in both RATE-seq data and the data generated by Presnyak *et al.* (2015), who used a RNA polymerase II temperature sensitive mutant for transcriptional shut-off (Table S2). We also find evidence that the presence of multiple sequential proline codons, which slows translation elongation, results in faster mRNA degradation, further supporting an effect of translation elongation rates on mRNA degradation rates.

By studying genome-wide degradation rates, we find evidence that the GC3 content is correlated negatively with rates of mRNA degradation. Despite the comparatively small amount of variance explained by GC3, the effect is significant and consistent with an earlier study that found a positive correlation between synonymous A|T dinucleotides spanning codon boundaries and mRNA degradation rates (Carlini 2005). To validate this effect experimentally, we tested the effect of GC3 content on mRNA degradation using GFP-encoding transcripts that differ in GC3 content. Consistent with our genome-wide analysis, we found that decreasing the GC3 content resulted in increased mRNA degradation rates, and lowered steady-state abundances. We surmise that the effect is not related to overall GC content of transcripts, as the GC content of the first (GC1) and second (GC2) position in codons explains much less variance. However, the effect of GC3 on mRNA degradation rate appears to be independent of the effect of codon bias, as the four GFP transcripts investigated do not show a systematic difference in CAI or nTE. Recently, increased GC3 content has been suggested to decrease mRNA degradation rates in human lymphoblastoid cells, and possibly explain variation in mRNA degradation rates between individuals (Duan *et al.* 2013). Thus, the relationship between GC3 content and mRNA degradation rates may be widely conserved in eukaryotes.

### Conclusion

Our study shows that genome-wide variation in mRNA degradation rates is best explained by a combination of different transcript features, as suggested more than two decades ago (Caponigro *et al.* 1993). Many of the factors that affect genome-wide patterns of mRNA degradation rates are related to protein production, highlighting the close relationship between mRNA degradation and translation. Our statistical analysis and experimental validation confirm the effect of ribosome density and GC3 content on mRNA degradation rates. Whereas mutation of a start codon likely changes translation initiation rates, modifying GC3 within a transcript is likely to alter translation elongation rates. Thus, both translation initiation and elongation rates may impact mRNA decay rates, but perhaps through different degradation pathways.

## Supplementary Material

Supplemental Material

## References

[bib1] Anderson J. S. Parker R. P., 1998 The 3′ to 5′ degradation of yeast mRNAs is a general mechanism for mRNA turnover that requires the SKI2 DEVH box protein and 3′ to 5′ exonucleases of the exosome complex. EMBO J. 17: 1497–1506. 9482746 10.1093/emboj/17.5.1497 PMC1170497

[bib2] Beelman C. A. Parker R., 1994 Differential effects of translational inhibition in cis and in trans on the decay of the unstable yeast MFA2 mRNA. J. Biol. Chem. 269: 9687–9692. 8144558

[bib3] Beelman C. A. Stevens A. Caponigro G. LaGrandeur T. E. Hatfield L., 1996 An essential component of the decapping enzyme required for normal rates of mRNA turnover. Nature 382: 642–646. 8757137 10.1038/382642a0

[bib4] Belle A. Tanay A. Bitincka L. Shamir R. O’Shea E. K., 2006 Quantification of protein half-lives in the budding yeast proteome. Proc. Natl. Acad. Sci. USA 103: 13004–13009. 16916930 10.1073/pnas.0605420103 PMC1550773

[bib5] Caponigro G. Muhlrad D. Parker R., 1993 A small segment of the MAT alpha 1 transcript promotes mRNA decay in *Saccharomyces cerevisiae*: a stimulatory role for rare codons. Mol. Cell. Biol. 13: 5141–5148. 8355674 10.1128/mcb.13.9.5141-5148.1993 PMC360202

[bib6] Carlini D. B., 2005 Context-dependent codon bias and messenger RNA longevity in the yeast transcriptome. Mol. Biol. Evol. 22: 1403–1411. 15772378 10.1093/molbev/msi135

[bib7] Chen C. Y. Gherzi R. Ong S. E. Chan E. L. Raijmakers R., 2001 AU binding proteins recruit the exosome to degrade ARE-containing mRNAs. Cell 107: 451–464. 11719186 10.1016/s0092-8674(01)00578-5

[bib8] Decker C. J. Parker R., 1993 A turnover pathway for both stable and unstable mRNAs in yeast: evidence for a requirement for deadenylation. Genes Dev. 7: 1632–1643. 8393418 10.1101/gad.7.8.1632

[bib9] Dressaire C. Picard F. Redon E. Loubière P. Queinnec I., 2013 Role of mRNA stability during bacterial adaptation. PLoS One 8: e59059. 23516597 10.1371/journal.pone.0059059 PMC3596320

[bib10] Duan J. Shi J. Ge X. Dölken L. Moy W., 2013 Genome-wide survey of interindividual differences of RNA stability in human lymphoblastoid cell lines. Sci. Rep. 3: 1318. 23422947 10.1038/srep01318 PMC3576867

[bib11] Eck S. Stephan W., 2008 Determining the relationship of gene expression and global mRNA stability in *Drosophila melanogaster* and *Escherichia coli* using linear models. Gene 424: 102–107. 18755255 10.1016/j.gene.2008.07.033

[bib12] Edri S. Tuller T., 2014 Quantifying the effect of ribosomal density on mRNA stability. PLoS One 9: e102308. 25020060 10.1371/journal.pone.0102308 PMC4096589

[bib13] Feng L. Niu D.-K., 2007 Relationship between mRNA stability and length: an old question with a new twist. Biochem. Genet. 45: 131–137. 17221301 10.1007/s10528-006-9059-5

[bib14] García-Martínez J. Aranda A. Pérez-Ortín J. E., 2004 Genomic run-on evaluates transcription rates for all yeast genes and identifies gene regulatory mechanisms. Mol. Cell 15: 303–313. 15260981 10.1016/j.molcel.2004.06.004

[bib15] Gardin J. Yeasmin R. Yurovsky A. Cai Y. Skiena S., 2014 Measurement of average decoding rates of the 61 sense codons in vivo. eLife 3. 10.7554/eLife.03735 PMC4371865 25347064

[bib16] Garí E. Piedrafita L. Aldea M. Herrero E., 1997 A set of vectors with a tetracycline-regulatable promoter system for modulated gene expression in *Saccharomyces cerevisiae*. Yeast 13: 837–848. 9234672 10.1002/(SICI)1097-0061(199707)13:9<837::AID-YEA145>3.0.CO;2-T

[bib17] Geisberg J. V. Moqtaderi Z. Fan X. Ozsolak F. Struhl K., 2014 Global analysis of mRNA isoform half-lives reveals stabilizing and destabilizing elements in yeast. Cell 156: 812–824. 24529382 10.1016/j.cell.2013.12.026 PMC3939777

[bib18] Ghaemmaghami S. Huh W.-K. Bower K. Howson R. W. Belle A., 2003 Global analysis of protein expression in yeast. Nature 425: 737–741. 14562106 10.1038/nature02046

[bib19] Grigull J. Mnaimneh S. Pootoolal J. Robinson M. D. Hughes T. R., 2004 Genome-wide analysis of mRNA stability using transcription inhibitors and microarrays reveals posttranscriptional control of ribosome biogenesis factors. Mol. Cell. Biol. 24: 5534–5547. 15169913 10.1128/MCB.24.12.5534-5547.2004 PMC419893

[bib20] Hager G. L. McNally J. G. Misteli T., 2009 Transcription dynamics. Mol. Cell 35: 741–753. 19782025 10.1016/j.molcel.2009.09.005 PMC6326382

[bib21] Hogan D. J. Riordan D. P. Gerber A. P. Herschlag D. Brown P. O., 2008 Diverse RNA-binding proteins interact with functionally related sets of RNAs, suggesting an extensive regulatory system. PLoS Biol. 6: e255. 18959479 10.1371/journal.pbio.0060255 PMC2573929

[bib22] Hong J. Gresham D., 2014 Molecular specificity, convergence and constraint shape adaptive evolution in nutrient-poor environments. PLoS Genet. 10: e1004041. 24415948 10.1371/journal.pgen.1004041 PMC3886903

[bib23] Ingolia N. T. Ghaemmaghami S. Newman J. R. S. Weissman J. S., 2009 Genome-wide analysis in vivo of translation with nucleotide resolution using ribosome profiling. Science 324: 218–223. 19213877 10.1126/science.1168978 PMC2746483

[bib24] Jing Q. Huang S. Guth S. Zarubin T. Motoyama A., 2005 Involvement of microRNA in AU-rich element-mediated mRNA instability. Cell 120: 623–634. 15766526 10.1016/j.cell.2004.12.038

[bib25] Kolitz S. E. Takacs J. E. Lorsch J. R., 2009 Kinetic and thermodynamic analysis of the role of start codon/anticodon base pairing during eukaryotic translation initiation. RNA 15: 138–152. 19029312 10.1261/rna.1318509 PMC2612769

[bib26] Kudla G. Lipinski L. Caffin F. Helwak A. Zylicz M., 2006 High guanine and cytosine content increases mRNA levels in mammalian cells. PLoS Biol. 4: e180. 16700628 10.1371/journal.pbio.0040180 PMC1463026

[bib27] Kudla G. Murray A. W. Tollervey D. Plotkin J. B., 2009 Coding-sequence determinants of gene expression in *Escherichia coli*. Science 324: 255–258. 19359587 10.1126/science.1170160 PMC3902468

[bib28] Lipson D. Raz T. Kieu A. Jones D. R. Giladi E., 2009 Quantification of the yeast transcriptome by single-molecule sequencing. Nat. Biotechnol. 27: 652–658. 19581875 10.1038/nbt.1551

[bib29] Merhi A. Gérard N. Lauwers E. Prévost M. André B., 2011 Systematic mutational analysis of the intracellular regions of yeast Gap1 permease. PLoS One 6: e18457. 21526172 10.1371/journal.pone.0018457 PMC3079708

[bib30] Miller C. Schwalb B. Maier K. Schulz D. Dümcke S., 2011 Dynamic transcriptome analysis measures rates of mRNA synthesis and decay in yeast. Mol. Syst. Biol. 7: 458. 21206491 10.1038/msb.2010.112 PMC3049410

[bib31] Muhlrad D. Parker R., 1992 Mutations affecting stability and deadenylation of the yeast MFA2 transcript. Genes Dev. 6: 2100–2111. 1427074 10.1101/gad.6.11.2100

[bib32] Muhlrad D. Decker C. J. Parker R., 1995 Turnover mechanisms of the stable yeast PGK1 mRNA. Mol. Cell. Biol. 15: 2145–2156. 7891709 10.1128/mcb.15.4.2145 PMC230442

[bib33] Munchel S. E. Shultzaberger R. K. Takizawa N. Weis K., 2011 Dynamic profiling of mRNA turnover reveals gene-specific and system-wide regulation of mRNA decay. Mol. Biol. Cell 22: 2787–2795. 21680716 10.1091/mbc.E11-01-0028 PMC3145553

[bib34] Nagalakshmi U. Wang Z. Waern K. Shou C. Raha D., 2008 The transcriptional landscape of the yeast genome defined by RNA sequencing. Science 320: 1344–1349. 18451266 10.1126/science.1158441 PMC2951732

[bib35] Narsai R. Howell K. A. Millar A. H. O’Toole N. Small I., 2007 Genome-wide analysis of mRNA decay rates and their determinants in *Arabidopsis thaliana*. Plant Cell 19: 3418–3436. 18024567 10.1105/tpc.107.055046 PMC2174890

[bib36] Neymotin B. Athanasiadou R. Gresham D., 2014 Determination of in vivo RNA kinetics using RATE-seq. RNA 20: 1645–1652. 25161313 10.1261/rna.045104.114 PMC4174445

[bib37] Nonet M. Scafe C. Sexton J. Young R., 1987 Eucaryotic RNA polymerase conditional mutant that rapidly ceases mRNA synthesis. Mol. Cell. Biol. 7: 1602–1611. 3299050 10.1128/mcb.7.5.1602-1611.1987 PMC365259

[bib38] Olivas W. Parker R., 2000 The Puf3 protein is a transcript-specific regulator of mRNA degradation in yeast. EMBO J. 19: 6602–6611. 11101532 10.1093/emboj/19.23.6602 PMC305854

[bib39] Parker R., 2012 RNA degradation in *Saccharomyces cerevisiae*. Genetics 191: 671–702. 22785621 10.1534/genetics.111.137265 PMC3389967

[bib40] Parker R. Jacobson A., 1990 Translation and a 42-nucleotide segment within the coding region of the mRNA encoded by the MAT alpha 1 gene are involved in promoting rapid mRNA decay in yeast. Proc. Natl. Acad. Sci. USA 87: 2780–2784. 2181450 10.1073/pnas.87.7.2780 PMC53774

[bib41] Pechmann S. Frydman J., 2013 Evolutionary conservation of codon optimality reveals hidden signatures of cotranslational folding. Nat. Struct. Mol. Biol. 20: 237–243. 23262490 10.1038/nsmb.2466 PMC3565066

[bib42] Pelechano V. Chávez S. Pérez-Ortín J. E., 2010 A complete set of nascent transcription rates for yeast genes. PLoS One 5: e15442. 21103382 10.1371/journal.pone.0015442 PMC2982843

[bib43] Plotkin J. B. Kudla G., 2011 Synonymous but not the same: the causes and consequences of codon bias. Nat. Rev. Genet. 12: 32–42. 21102527 10.1038/nrg2899 PMC3074964

[bib44] Presnyak V. Alhusaini N. Chen Y.-H. Martin S. Morris N., 2015 Codon optimality is a major determinant of mRNA stability. Cell 160: 1111–1124. 25768907 10.1016/j.cell.2015.02.029 PMC4359748

[bib57] R Core Team 2013 R: A language and environment for statistical computing. R Foundation for Statistical Computing, Vienna, Austria URL http://www.R-project.org/.

[bib45] Rabani M. Levin J. Z. Fan L. Adiconis X. Raychowdhury R., 2011 Metabolic labeling of RNA uncovers principles of RNA production and degradation dynamics in mammalian cells. Nat. Biotechnol. 29: 436–442. 21516085 10.1038/nbt.1861 PMC3114636

[bib46] Selinger D. W. Saxena R. M. Cheung K. J. Church G. M. Rosenow C., 2003 Global RNA half-life analysis in *Escherichia coli* reveals positional patterns of transcript degradation. Genome Res. 13: 216–223. 12566399 10.1101/gr.912603 PMC420366

[bib47] Shalem O. Dahan O. Levo M. Martinez M. R. Furman I., 2008 Transient transcriptional responses to stress are generated by opposing effects of mRNA production and degradation. Mol. Syst. Biol. 4: 223. 18854817 10.1038/msb.2008.59 PMC2583085

[bib48] Sharp P. M. Li W. H., 1987 The Codon Adaptation Index—a measure of directional synonymous codon usage bias, and its potential applications. Nucleic Acids Res. 15: 1281–1295. 3547335 10.1093/nar/15.3.1281 PMC340524

[bib49] Shaw G. Kamen R., 1986 A conserved AU sequence from the 3′ untranslated region of GM-CSF mRNA mediates selective mRNA degradation. Cell 46: 659–667. 3488815 10.1016/0092-8674(86)90341-7

[bib50] Thomsen S. Anders S. Janga S. C. Huber W. Alonso C. R., 2010 Genome-wide analysis of mRNA decay patterns during early *Drosophila* development. Genome Biol. 11: R93. 20858238 10.1186/gb-2010-11-9-r93 PMC2965385

[bib51] Wang Y. Liu C. L. Storey J. D. Tibshirani R. J. Herschlag D., 2002 Precision and functional specificity in mRNA decay. Proc. Natl. Acad. Sci. USA 99: 5860–5865. 11972065 10.1073/pnas.092538799 PMC122867

[bib52] Wisdom R. Lee W., 1991 The protein-coding region of c-myc mRNA contains a sequence that specifies rapid mRNA turnover and induction by protein synthesis inhibitors. Genes Dev. 5: 232–243. 1995415 10.1101/gad.5.2.232

[bib53] Wishart J. A. Hayes A. Wardleworth L. Zhang N. Oliver S. G., 2005 Doxycycline, the drug used to control the tet-regulatable promoter system, has no effect on global gene expression in *Saccharomyces cerevisiae*. Yeast 22: 565–569. 15942933 10.1002/yea.1225

[bib54] Yang E. van Nimwegen E. Zavolan M. Rajewsky N. Schroeder M., 2003 Decay rates of human mRNAs: correlation with functional characteristics and sequence attributes. Genome Res. 13: 1863–1872. 12902380 10.1101/gr.1272403 PMC403777

[bib55] Yun D.-F. Sherman F., 1996 Degradation of CYC1 mRNA in the yeast *Saccharomyces cerevisiae* does not require translation. Proc. Natl. Acad. Sci. USA 93: 8895–8900. 8799124 10.1073/pnas.93.17.8895 PMC38565

[bib56] Zenklusen D. Larson D. R. Singer R. H., 2008 Single-RNA counting reveals alternative modes of gene expression in yeast. Nat. Struct. Mol. Biol. 15: 1263–1271. 19011635 10.1038/nsmb.1514 PMC3154325

