# Supplementary material for: Multiple Transcript Properties Related to Translation Affect mRNA Degradation Rates in Saccharomyces cerevisiae
Source: G3 (Bethesda). 2016 Sep 13;6(11):3475–83. doi: 10.1534/g3.116.032276 (PMC5100846; doi:10.1534/g3.116.032276)
Supplement: Supplemental Material [file supp_6_11_3475__index.html]

Multiple Transcript Properties Related to Translation Affect mRNA Degradation Rates in Saccharomyces cerevisiae — Supplemental Material 

# Multiple Transcript Properties Related to Translation Affect mRNA Degradation Rates in *Saccharomyces cerevisiae*

## Supplemental Material for Neymotin, Ettore, and Gresham, 2016

**Files in this Data Supplement:**

- Figure S1 - Multiple predictors best explain variation in mRNA degradation. (.pdf, 253 KB)
- Figure S2 - Predictors of mRNA degradation variation are highly correlated. (.pdf, 179 KB)
- Figure S3 - mRNA degradation rate decreases with increased ribosome density. (.pdf, 4 MB)
- Figure S4 - GFP1 and GFP2 have similar mRNA degradation kinetics. (.pdf, 104 KB)
- Figure S5 - Effect of mutated start codon on GFP protein. (.pdf, 143 KB)
- Figure S6 - Diagnostics of the model fit. (.pdf, 2 MB)
- Figure S7 - Adjusted R2 when each individual predictor is removed from the final model. (.pdf, 117 KB)
- Figure S8 - Significant differences in transcript features for genes that fit the model poorly. (.pdf, 162 KB)
- Table S1 - Matrix with all parameters included in regression analysis. (.xlsx, 2 MB)
- Table S2 - Matrix with p-?value, adjusted R2, and Pearson correlation for each predictor and reported mRNA degradation rate. (.xlsx, 13 KB)
- Table S3 - Data for qPCR experiments. (.xlsx, 55 KB)
