# Supplementary material for: Multiple Transcript Properties Related to Translation Affect mRNA Degradation Rates in Saccharomyces cerevisiae
Source: G3 (Bethesda). 2016 Sep 13;6(11):3475–83. doi: 10.1534/g3.116.032276 (PMC5100846; doi:10.1534/g3.116.032276)
Supplement: Supplemental Material [file supp_g3.116.032276_FigureS1.pdf]

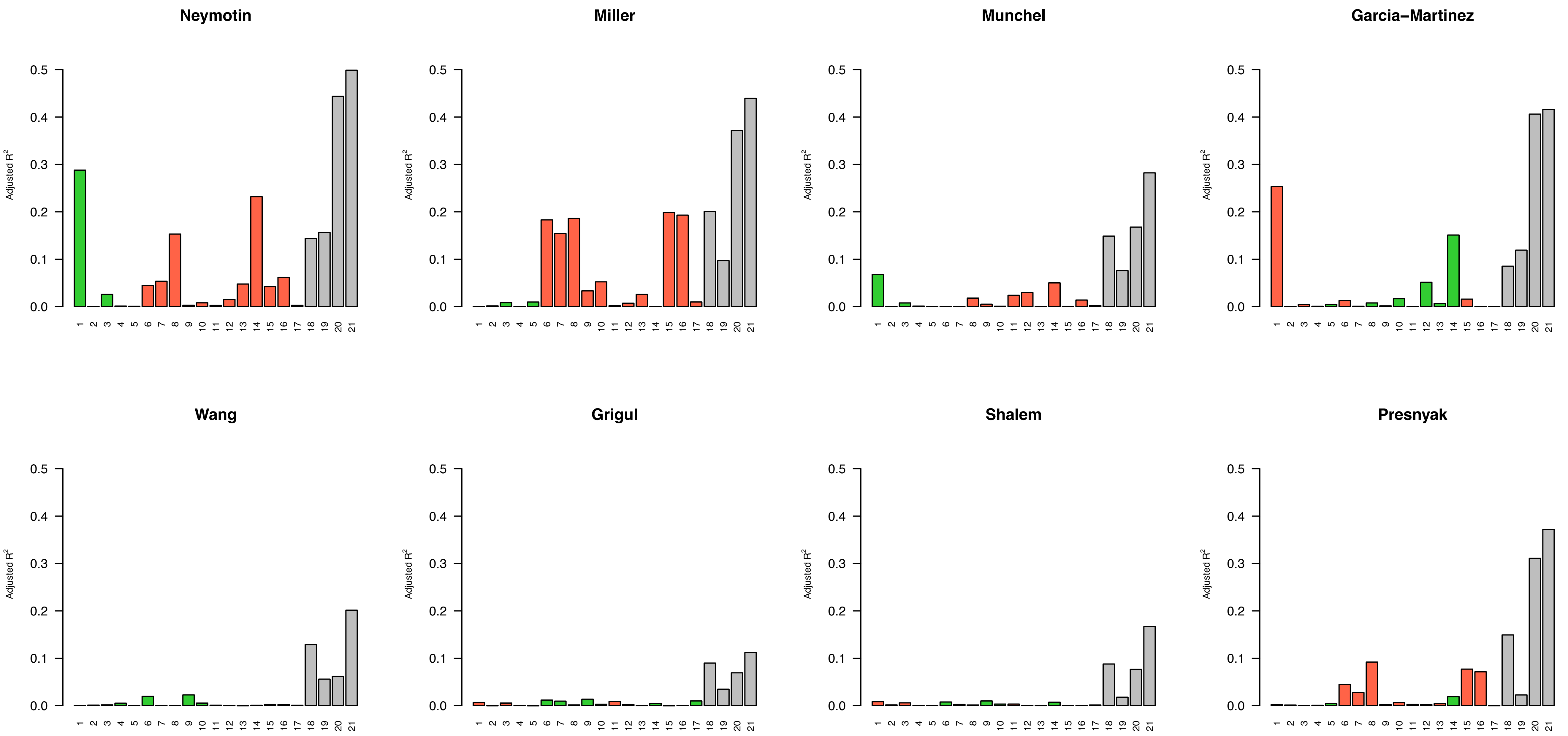

Figure S1. Multiple predictors best explain variation in mRNA degradation. We show the adjusted R2 for individual predictors in each model. Predictors are as follows: 1=Coding length, 2=3'UTR length, 3=5'UTR length, 4=5'UTR GC content, 5=3'UTR GC content, 6=mRNA abundance, 7=Protein per cell, 8=Ribosome density, 9=Transcription rate, 10=GC1, 11=GC2, 12=GC3, 13=Protein half-life, 14=deltaG, 15=CAI, 16=nTE,17=Protein per mRNA, 18=GO, 19=RNABP, 20=model with continuous variables, and 21=model with continuous variables +GO +RNABP.
