# Supplementary material for: Multiple Transcript Properties Related to Translation Affect mRNA Degradation Rates in Saccharomyces cerevisiae
Source: G3 (Bethesda). 2016 Sep 13;6(11):3475–83. doi: 10.1534/g3.116.032276 (PMC5100846; doi:10.1534/g3.116.032276)
Supplement: Supplemental Material [file supp_g3.116.032276_FigureS2.pdf]

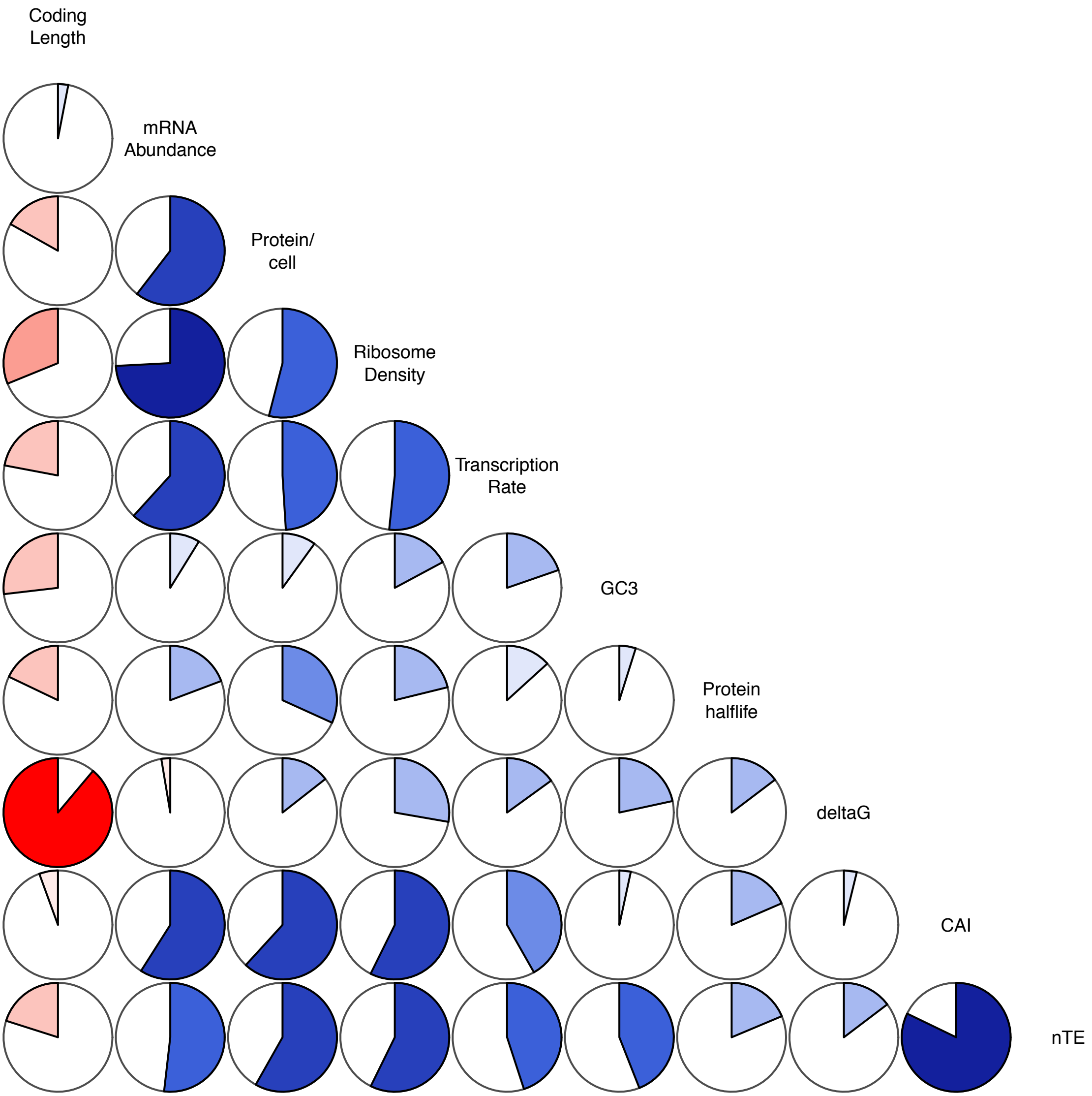

Figure S2. Predictors of mRNA degradation variation are highly correlated. Pie charts indicate the strength of the Pearson correlation (values vary between 0 and 1) by the size of the colored pie. Blue is a positive correlation and red is a negative correlation.
