# Supplementary material for: Multiple Transcript Properties Related to Translation Affect mRNA Degradation Rates in Saccharomyces cerevisiae
Source: G3 (Bethesda). 2016 Sep 13;6(11):3475–83. doi: 10.1534/g3.116.032276 (PMC5100846; doi:10.1534/g3.116.032276)
Supplement: Supplemental Material [file supp_g3.116.032276_FigureS3.pdf]

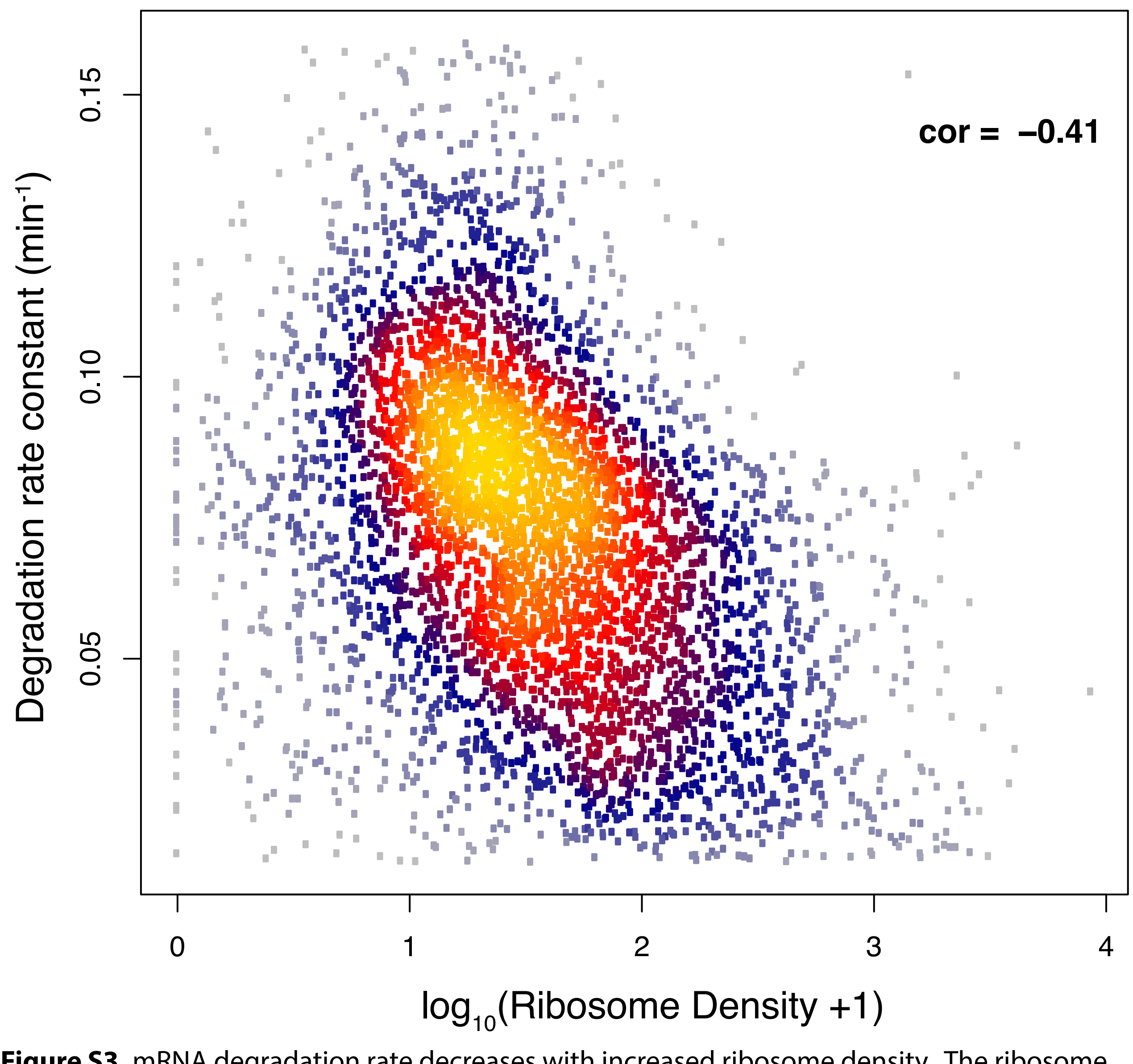

Figure S3. mRNA degradation rate decreases with increased ribosome density. The ribosome density of a transcript is inversely correlated with degradation rate, indicating that the more ribosomes associated with a transcript, the more stable the transcript.
