# Supplementary figures and images for: Multiple Transcript Properties Related to Translation Affect mRNA Degradation Rates in Saccharomyces cerevisiae
Source: G3 (Bethesda). 2016 Sep 13;6(11):3475–83. doi: 10.1534/g3.116.032276 (PMC5100846; doi:10.1534/g3.116.032276)

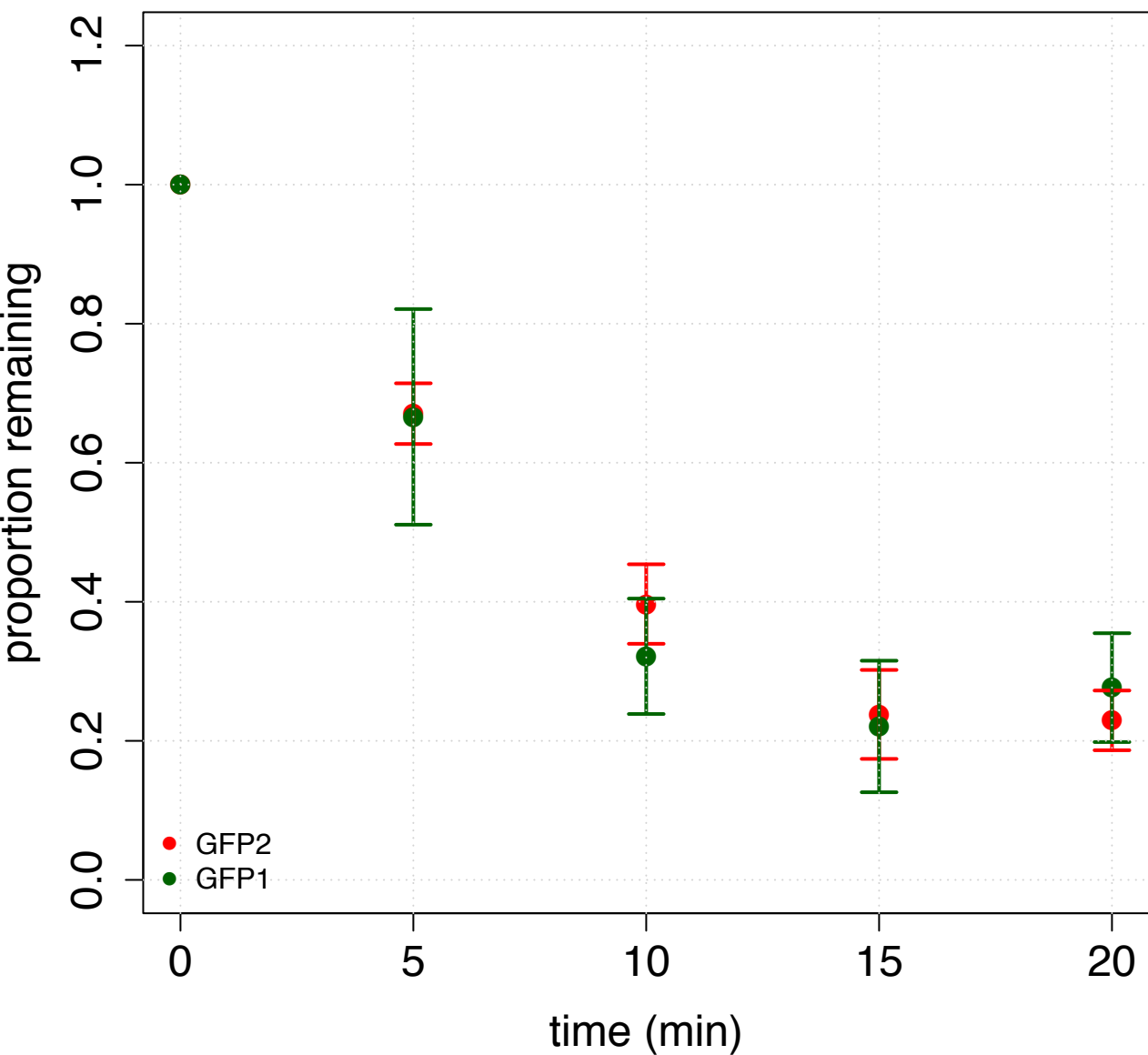

**Figure S4.** GFP1 and GFP2 have similar mRNA degradation kinetics.

Supplement: Supplemental Material [file supp_g3.116.032276_FigureS4.pdf]

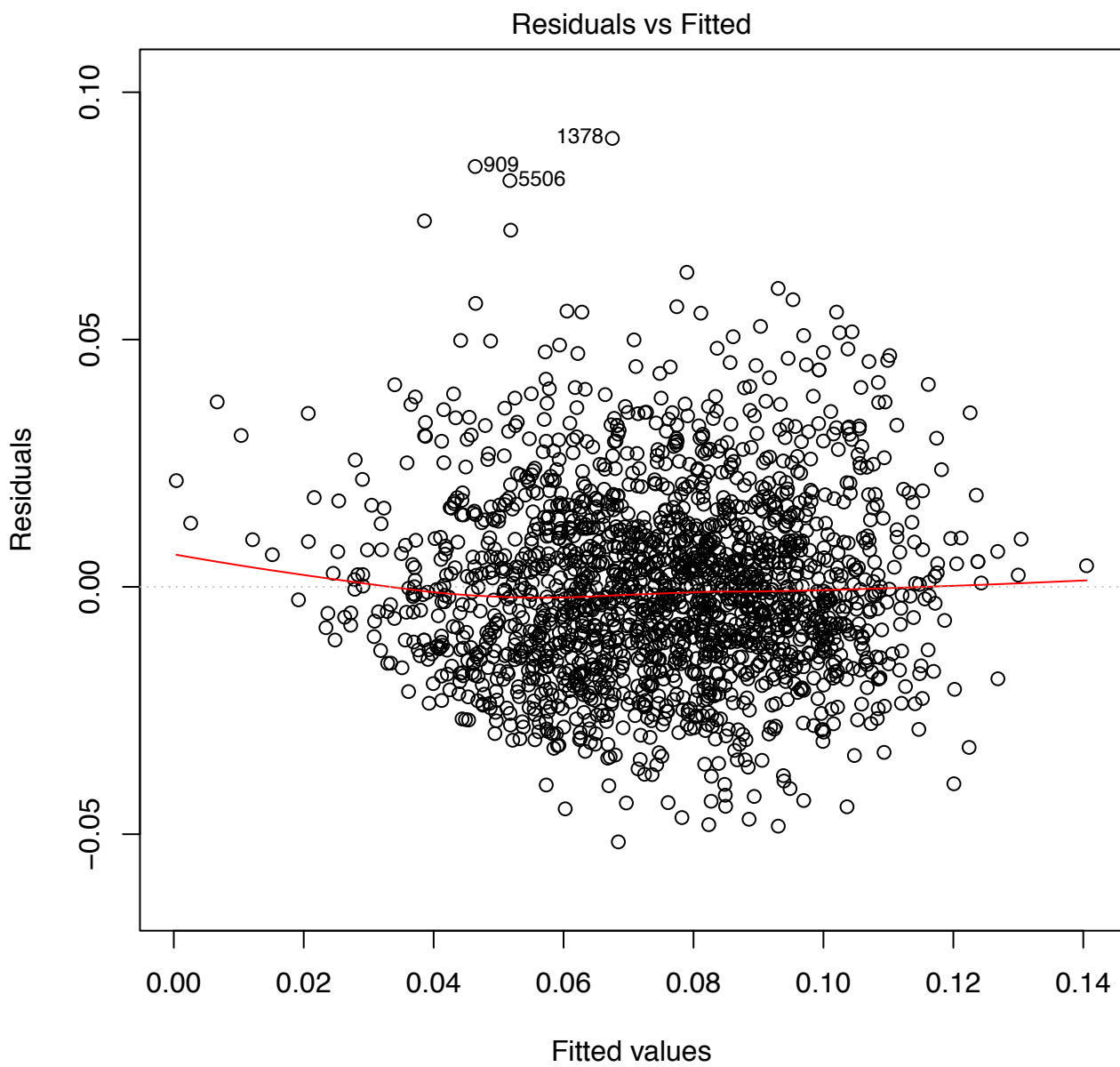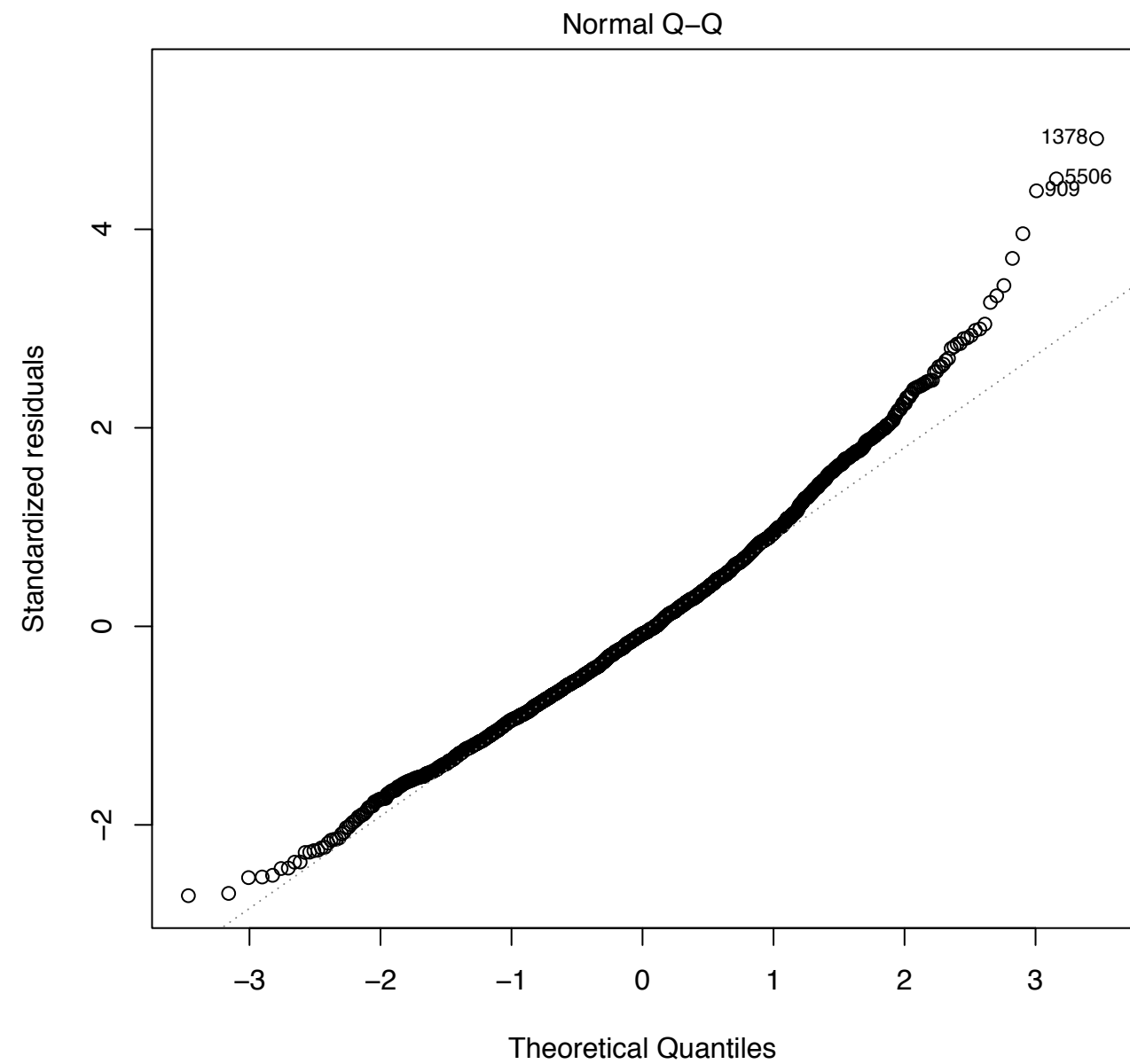

**Figure S6.** Diagnostics of the model fit.

Supplement: Supplemental Material [file supp_g3.116.032276_FigureS6.pdf]
