# Supplementary material for: Multiple Transcript Properties Related to Translation Affect mRNA Degradation Rates in Saccharomyces cerevisiae
Source: G3 (Bethesda). 2016 Sep 13;6(11):3475–83. doi: 10.1534/g3.116.032276 (PMC5100846; doi:10.1534/g3.116.032276)
Supplement: Supplemental Material [file supp_g3.116.032276_FigureS5.pdf]

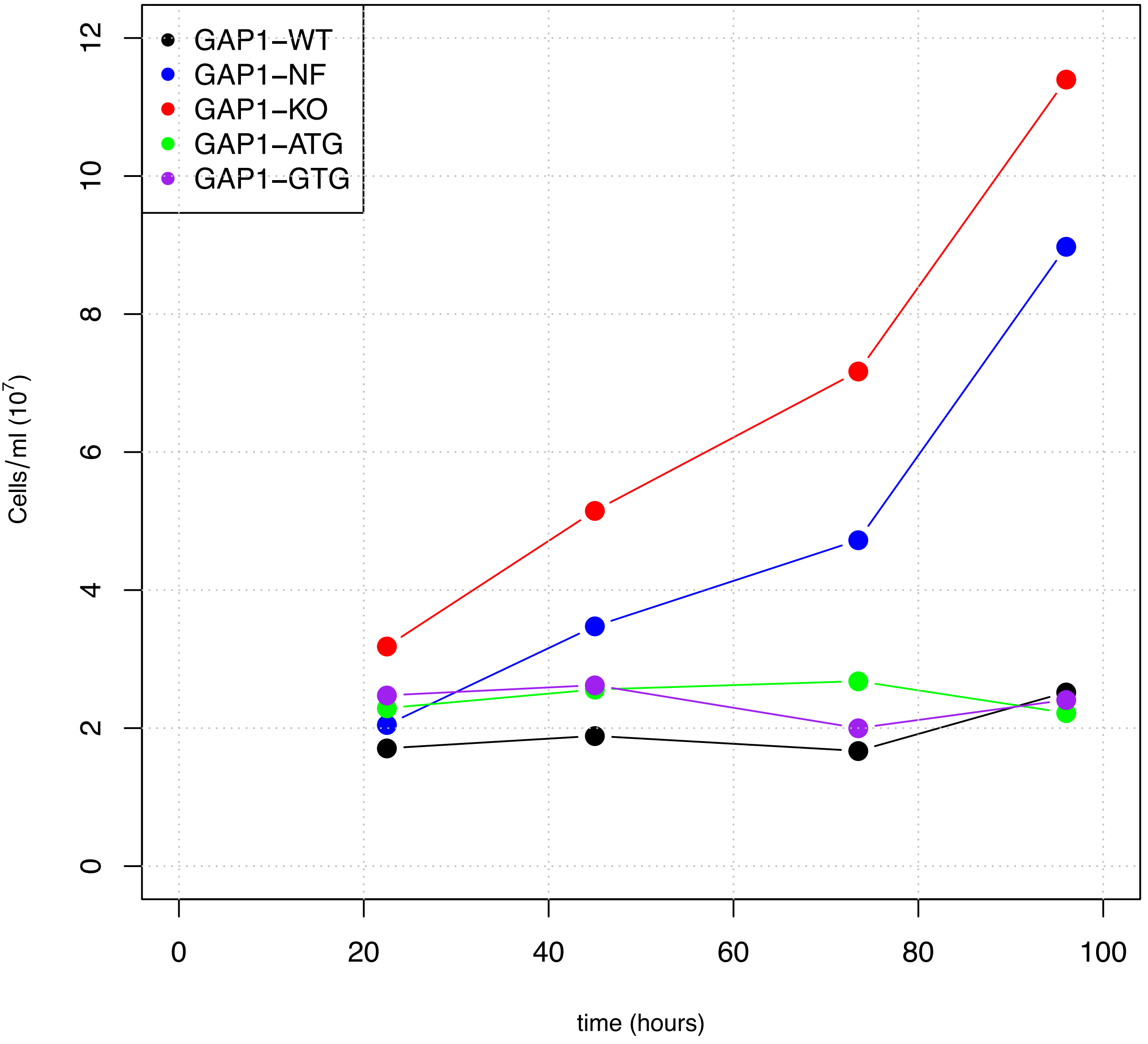

Figure S5. Effect of mutated start codon on GFP protein. Yeast cells were grown in minimal media containing D-histidine and D-serine to assay for functional GAP1 protein. We show five strains: GAP1-WT = wild type strain, GAP1-KO = strain with complete knockout of coding sequence of GAP1, GAP1-NF= Strain containing non-functional GAP1, GAP1-ATG = GAP1-KO strain carrying plasmid containing GAP1 with normal start codon and GAP1-GTG = GAP1-KO strain carrying plasmid containing GAP1 with GTG in place of start codon.
