# Supplementary material for: Multiple Transcript Properties Related to Translation Affect mRNA Degradation Rates in Saccharomyces cerevisiae
Source: G3 (Bethesda). 2016 Sep 13;6(11):3475–83. doi: 10.1534/g3.116.032276 (PMC5100846; doi:10.1534/g3.116.032276)
Supplement: Supplemental Material [file supp_g3.116.032276_FigureS7.pdf]

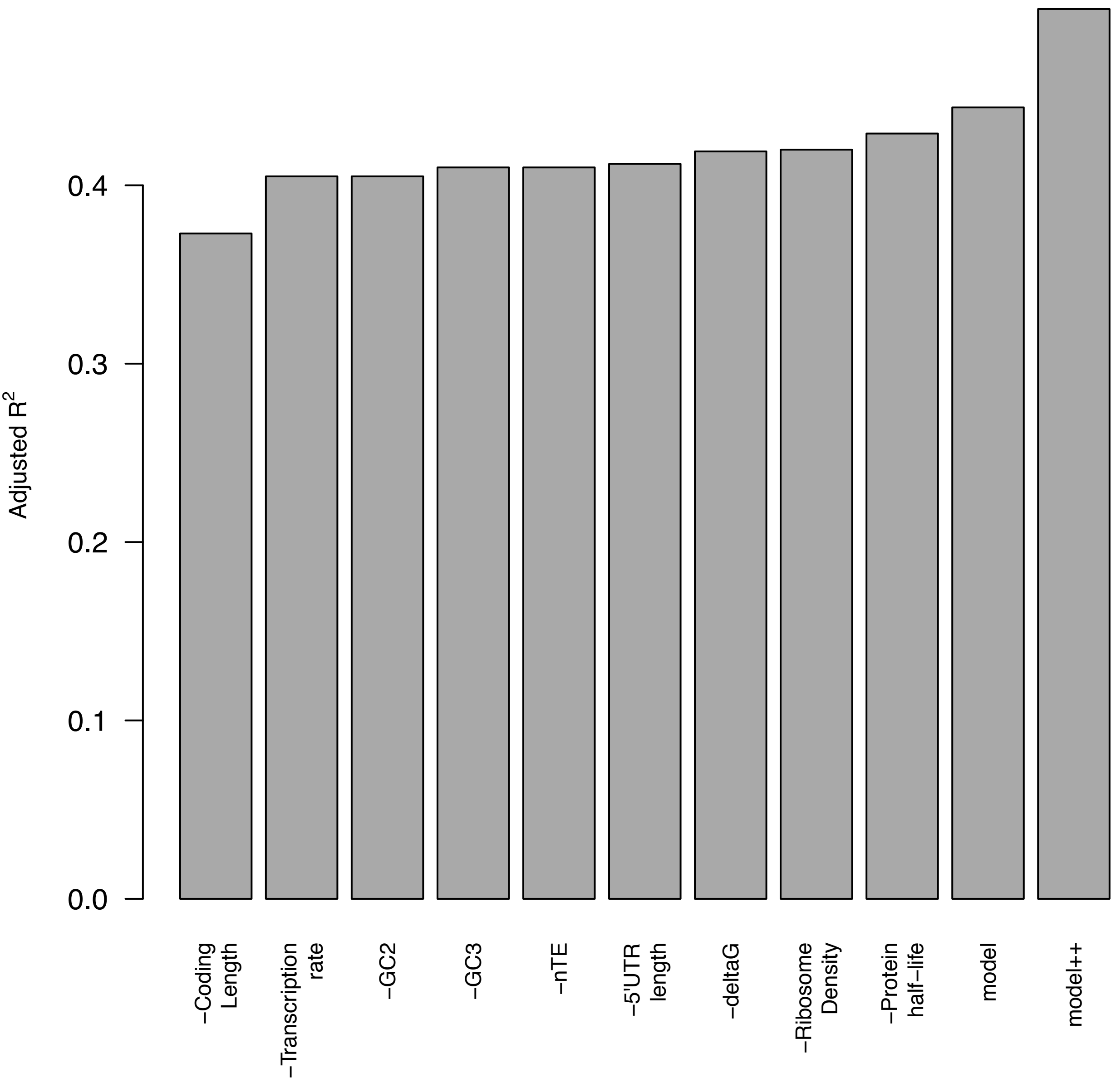

Figure S7. Adjusted R2 when each individual predictor is removed from the final model. The predictor removed is indicated with a minus in front of it. The adjusted R2 for full model is shown for reference.
