# Supplementary material for: Multiple Transcript Properties Related to Translation Affect mRNA Degradation Rates in Saccharomyces cerevisiae
Source: G3 (Bethesda). 2016 Sep 13;6(11):3475–83. doi: 10.1534/g3.116.032276 (PMC5100846; doi:10.1534/g3.116.032276)
Supplement: Supplemental Material [file supp_g3.116.032276_FigureS8.pdf]

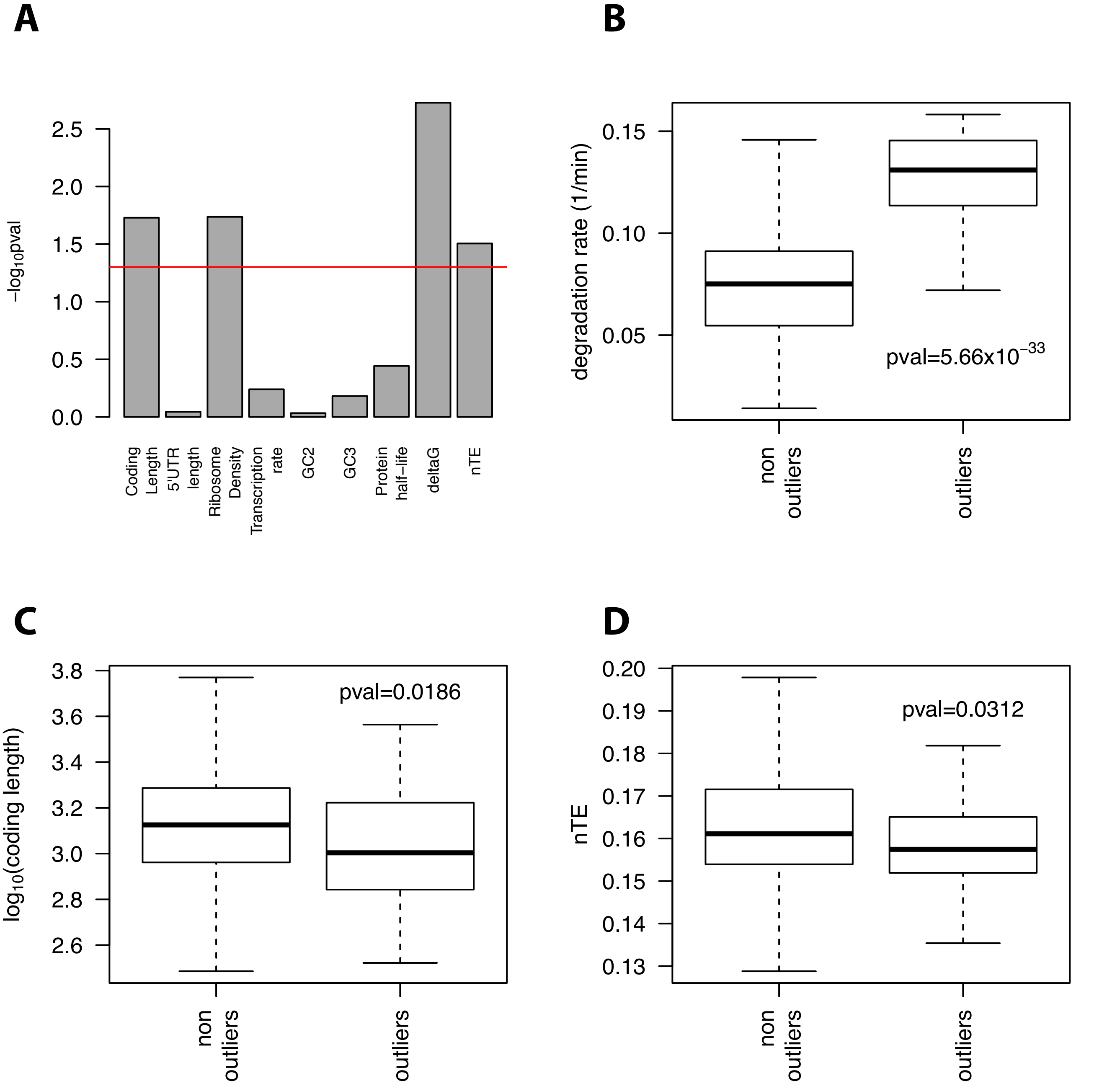


**Figure S8.** Significant differences in transcript features for genes that fit the model poorly. **A**) p-values from two sample t-tests for differences in transcript properties between outliers and non-outliers for all predictors in multiple regression model. The red line indicates a p-value of 0.05. **B**) The outlier transcripts that fit the model poorly tend to degrade faster. The p-value indicates the result of a two sample t-test testing whether the average decay rate is significantly different between the two groups of transcripts. **C**) The outlier transcripts that fit the model poorly tend to be shorter. The p-value indicates the result of a two sample t-test between the two groups of genes. **D**) The outlier transcripts that fit the model poorly tend to be less translationally efficient. The p-value indicates the result of a two sample t-test between the two groups of genes.
